# Children's Hospitals in England

**Published:** 1886-11-20

**Authors:** 


### CHILDREN'S HOSPITALS IN ENGLAND.

Miss Jessy Dromgole writes from The Evergreens, Blenheim Road, Wood Green, N.: "I have been informed that you will be kind enough to grant any information coming within the province of your paper; can you therefore oblige me by sending me a list of Children's Hospitals throughout England?"

⁂ The desired information will be found in the pages of a little book called *Help in Sickness* published by Kegan Paul, Trench and Co., Paternoster Square, E.C., price 1*s*. 6*d*. They are too numerous to give in detail here.—Ed. T. H.

---

The Literary Prize is unavoidably held over until next week, the Puzzle Editor not yet having made his decision.

